# Impact of Superoxide Dismutase Mimetic AEOL 10150 on the Endothelin System of Fischer 344 Rats

**DOI:** 10.1371/journal.pone.0151810

**Published:** 2016-03-18

**Authors:** Devi Ganesh, Prem Kumarathasan, Errol M. Thomson, Carly St-Germain, Erica Blais, James Crapo, Renaud Vincent

**Affiliations:** 1 Department of Biochemistry, Microbiology and Immunology, Faculty of Medicine, University of Ottawa, Ottawa, Ontario, Canada; 2 Environmental Health Science and Research Bureau, Environmental and Radiation Health Sciences Directorate, Healthy Environments and Consumer Safety Branch, Health Canada, Ottawa, Ontario, Canada; 3 National Jewish Health, Denver, Colorado, United States of America; University of Florida, UNITED STATES

## Abstract

Endothelin-1 is a potent vasoconstrictor and mitogenic peptide involved in the regulation of vasomotor tone and maintenance of blood pressure. Oxidative stress activates the endothelin system, and is implicated in pulmonary and cardiovascular diseases including hypertension, congestive heart failure, and atherosclerosis. Superoxide dismutase mimetics designed with the aim of treating diseases that involve reactive oxygen species in their pathophysiology may exert a hypotensive effect, but effects on the endothelin system are unknown. Our objective was to determine the effect of the superoxide dismutase mimetic AEOL 10150 on the basal endothelin system *in vivo*. Male Fischer-344 rats were injected subcutaneously with 0, 2 or 5 mg/kg body weight of AEOL 10150 in saline. Plasma oxidative stress markers and endothelins (bigET-1, ET-1, ET-2, ET-3) as well as lung and heart endothelin/nitric oxide system gene expressions were measured using HPLC-Coularray, HPLC-Fluorescence and RT-PCR respectively. AEOL 10150 reduced (p<0.05) the circulating levels of isoprostane (-25%) and 3-nitrotyrosine (-50%) measured in plasma 2h and 24h after treatment, confirming delivery of a physiologically-relevant dose and the potent antioxidant activity of the drug. The reduction in markers of oxidative stress coincided with sustained 24h decrease (p<0.05) of plasma levels of ET-1 (-50%) and ET-3 (-10%). Expression of preproET-1 and endothelin converting enzyme-1 mRNA were not altered significantly in the lungs. However preproET-1 (not significant) and ECE-1 mRNA (p<0.05) were increased (10–25%) in the heart. Changes in the lungs included decrease (p<0.05) of mRNA for the ET-1 clearance receptor ET_B_ and the vasoconstriction-signaling ET_A_ receptor (-30%), and an early surge of inducible nitric oxide synthase expression followed by sustained decrease (-40% after 24 hours). The results indicate that interception of the endogenous physiological flux of reactive nitrogen species and reactive oxygen species in rats impacts the endothelin/nitric oxide system, supporting a homeostatic relationship between those systems.

## Introduction

Endothelins (ETs) are potent vasoactive peptides that play important roles in homeostatic control of vessel tone in healthy individuals as well as being implicated in a number of disease states [1]. Observations of ET system dysfunction observed in both experimental animal models and clinical studies of cardiovascular [2–4] and pulmonary [5–7] disease have led to the identification of the endothelin system as a therapeutic target. Patients with pulmonary artery hypertension display increased ET-1 plasma levels and treatment with ET receptor antagonists in experimental models and clinical trials have shown a reduction in pulmonary vascular pressure, right ventricular hypertrophy and pulmonary artery wall thickening [8, 9]. Both selective and non-selective ET receptor antagonists (ambrisentan, and bosentan, respectively) are currently employed for treatment of pulmonary artery hypertension [9, 10]. Endothelin-1 is also a pro-fibrotic factor, and bosentan has been approved for treatment of the fibrosis disease scleroderma [9]. Although promising in vivo data suggest that treatment with ET receptor antagonists can produce beneficial outcomes in models of cardiovascular disease [11–13], results in clinical trials have proven less definitive.

Oxidative stress is implicated in many disease states, and has been shown to play a role in regulating ET levels [14–17], which in turn can modulate production of reactive oxygen species [18–20]. For example, oxidative stress-induced increases in preproET-1 mRNA were abolished by superoxide dismutase [14]. Conversely, production of reactive oxygen species following treatment of fetal pulmonary artery smooth muscle cells with ET-1 was attenuated by antioxidant treatment [19]. Clinical studies have shown a positive correlation of antioxidant levels and ET-1 expression [21, 22]. Taken together, these studies indicate that oxidative stress activates the endothelinergic system, an effect that can be mitigated by antioxidants.

The importance of oxidative stress in disease processes has led to the development of antioxidant therapeutics. Metalloporphyrin complexes mimic the biologic activity of superoxide dismutase, including scavenging of superoxide, H_2_O_2_, peroxynitrite, and lipid peroxyl radicals [23, 24], and can pharmacologically augment natural antioxidant defenses [25]. The superoxide dismutase mimetic manganese (III) mesotetrakis (di-N-ethylimidazole) porphyrin AEOL 10150 is a low molecular weight, synthetic, redox-active, catalytic antioxidant that was shown in rodent models to attenuate expression of inflammatory genes in stroke [26] and to reduce tobacco smoke-induced inflammation and lung injury when delivered by intratracheal instillation before smoke exposure [27]. Recent studies demonstrate AEOL 10150 as an effective rescue treatment following exposure to chlorine gas and 2-chloroethyl ethyl sulfide through reduction in inflammation, lung injury, and oxidative stress [28, 29]. There is some evidence that metalloporphyrin catalytic antioxidants can exert hypotensive effects in rats [30], but effects on the ET system have not been examined.

The aim of this study was to characterize the impact of the superoxide dismutase mimetic AEOL 10150 on circulating ET peptide levels and expression of endothelin system genes. For this purpose, we chose to use an inbred normotensive healthy rat model (Fischer 344) since we were interested in the impact of AEOL 10150 on a healthy cardiovascular system. We treated these rats with AEOL 10150 and followed the oxidative stress levels and circulating endothelin profiles.

## Materials and Methods

### Animals

Pathogen-free Fischer-344 male rats (180–250g) were obtained from Charles River (St. Constant, Québec, Canada). The animals were housed in individual Plexiglas cages on wood-chip bedding under HEPA-filtered air and held to a 12h dark/light cycle. Food and water were provided ad libitum. All experimental protocols were reviewed and approved by the Animal Care Committee of Health Canada (Ottawa, Ontario, Canada).

### Subcutaneous Injections

AEOL 10150 (manganese (III) meso-tetrakis (di-N-ethylimidazole) porphyrin), a superoxide dismutase mimetic, is a stable, nontoxic, water soluble compound with a superoxide dismutase activity of ~5,000 units/g and catalase activity of approximately ~1% of purified bovine catalase (weight/weight basis). Animals were randomized initially prior to any treatment and were weighed. In this study AEOL 10150, a gift from Incara Pharmaceuticals (Research Triangle Park, NC, USA), was subcutaneously injected into animals at concentrations 0, 2 or 5 mg/kg body weight (BW) in 1 mL/kg BW of saline at 8am (n = 6 animals per group). Sample size was determined based on our previous work. The mode of drug administration was based on previous literature [31]. All treatments and assessments were randomized to avoid any bias.

### Biological Samples

Rats were anaesthetized at 2h or 24h following dosing with AEOL 10150 by administration of sodium pentobarbital (60 mg/kg, ip). Blood was collected from the abdominal aorta and combined with protease inhibitors (10 mg/mL ethylene diamine tetra acetic acid (EDTA) and 1.7 mg/mL phenyl methyl sulfonyl fluoride (PMSF) [32]. The animals were then exsanguinated, and the trachea was exposed and cannulated. The diaphragm was punctured to collapse the lungs, and the lungs were filled by intratracheal instillation of warm (37°C) calcium- and magnesium-free Dulbecco's phosphate buffered saline (Sigma Chemical Co., St. Louis, MO) at a ratio of 35 mL/kg BW. The saline was aspirated and re-injected twice more, and the bronchoalveolar lavage fluid was recovered. This lavage fluid was further used for total protein analysis as previously described [33]. The lungs and heart were recovered, flash frozen in liquid nitrogen, and stored at -80°C.

### Plasma Samples

Briefly, blood samples were centrifuged at 1448 x g for 10 min to obtain plasma. Plasma samples were recovered and vortexed with 50 μL of aqueous 0.1M DETPA solution and 50 μL of 0.3M BHT solution in isopropanol to prevent any post-mortem changes due to autoxidation. Two sets of plasma aliquots (250 μL) were used for 8-isoprostane and for protein oxidation, nitration marker analyses, while another set of aliquots was used for analysis of circulating endothelin levels.

### Plasma Isoprostane

Aliquots of plasma (250 μL) samples stabilized with DETPA and BHT were used for the 8-isoprostane (15(s)-8-iso-PGF2α)analysis. Plasma samples were purified initially using C18 cartridges and were analysed using a competitive enzyme immunoassay kit (Cayman Chemicals, Ann Arbor, Michigan, USA) following a procedure reported earlier [34].

### Plasma 3-Nitrotyrosine

Nitrative stress levels in rat plasma were measured by analysis of protein nitration product 3-nitrotyrosine using the HPLC-Coularray method described previously [35]. Here, 250 μL aliquots of plasma samples stabilized with DETPA and BHT were deproteinized by use of acidified acetone, evaporated to concentrate, clarified using molecular weight cut-off filters (30 kDa), dried under N_2_ flow and were reconstituted with 100 μL of acidified water prior to the analysis by the HPLC-Coularray method. Initial separation of analytes were carried out on a LC-18 reversed phase column (25 cm length, 4.6 mm id, 5μm particle size; Supelco, Oakville, ON) by isocratic elution using a citrate-acetate buffer (pH = 4.7) mobile phase containing OSA as the ion-pair reagent. Separated analytes were measured by coulometric array detection using a set of eight electrodes at different applied potentials.

### Plasma Endothelins

This procedure was conducted using a previously reported method [32]. Briefly, plasma aliquots (250 μL) were treated with 3,4-dichloroisocoumarin solution in isopropanol to prevent conversion of big ET-1 (BET-1) to ET-1 during sample processing. Samples were deproteinized by acidified acetone, evaporated to concentrate, and then cleaned up using molecular weight cut-off filters (30 kDa). Clarified samples were dried under a N_2_ flow and were reconstituted in phosphate buffered saline and were analyzed by a reversed phase HPLC-Fluorescence system. Initial separation of endothelin isoforms was carried out on a LC-318 column (25 cm length, 4.6 mm id, 5μm particle size; Supelco, Oakville, ON) by gradient elution using water-acetonitrile mobile phase [A-30% acetonitrile (aq); B-90% acetonitrile (aq)] with 0.19% of TFA used as the ion-pair reagent. Analytes were measured by fluorescence detection at excitation and emission wavelengths of 240 nm and 380 nm, respectively.

### Gene Expression

Real-time reverse transcription polymerase chain reaction (RT-PCR) was conducted using validated primer sets as previously described [36]. Double-desalted primers for β-actin, preproET-1, ECE-1, ET_A_ receptor, ET_B_ receptor, eNOS and iNOS were purchased from Invitrogen, Canada. Briefly, frozen lung and heart samples were homogenized in TRIzol reagent (Invitrogen Canada Inc., Burlington, Ontario, Canada), and total RNA was isolated according to the manufacturer’s instructions. RNA was quantified using the RiboGreen RNA Quantitation Reagent and Kit (Molecular Probes, Eugene, OR, USA), and total RNA was reverse transcribed using MuLV reverse transcriptase and random hexamers (Applied Biosystems, Mississauga, Ontario, Canada) according to the manufacturer’s instructions. Twenty nanograms cDNA was incubated with 25 μL iQ SYBR Green Supermix (Bio-Rad Laboratories (Canada) Ltd., Mississauga, Ontario, Canada) and 200 nM of each primer, and the reagent mixture was brought up to 50 μL with DNase/RNase-free water. All reactions were performed in duplicate on 96-well plates in a spectrofluorometric thermal cycler (iCycler iQ, Bio-Rad, Bio-Rad Laboratories (Canada) Ltd., Mississauga, Ontario, Canada). Uniform reaction conditions were confirmed as previously described [37]. PCR runs were initiated by incubation at 95°C for 3 min to activate the iTAQ polymerase followed by 40 cycles of 95°C for 15 s, the appropriate annealing temperature for 15 s (60°C for ET_B_ receptor and 62°C for all other genes), and 30 s at 72°C. Fluorescence was monitored at every cycle during the elongation step. A melt curve was conducted following each run to verify product purity. Expression was calculated relative to β-actin using the delta-Ct method [38], and expressed as fold change relative to control samples.

### Statistical Analyses

Statistical significance was assessed by 2-way ANOVA with AEOL (0, 2, 5 mg/kg BW) and TIME (2, 24h) as factors (n = 6 animals per group), followed by Holm-Sidak multiple comparison procedure to elucidate the pattern of significant effects (α = 0.05). Data are expressed as mean ± SEM.

## Results

### Oxidative and Nitrative Stress

**Fig 1:**
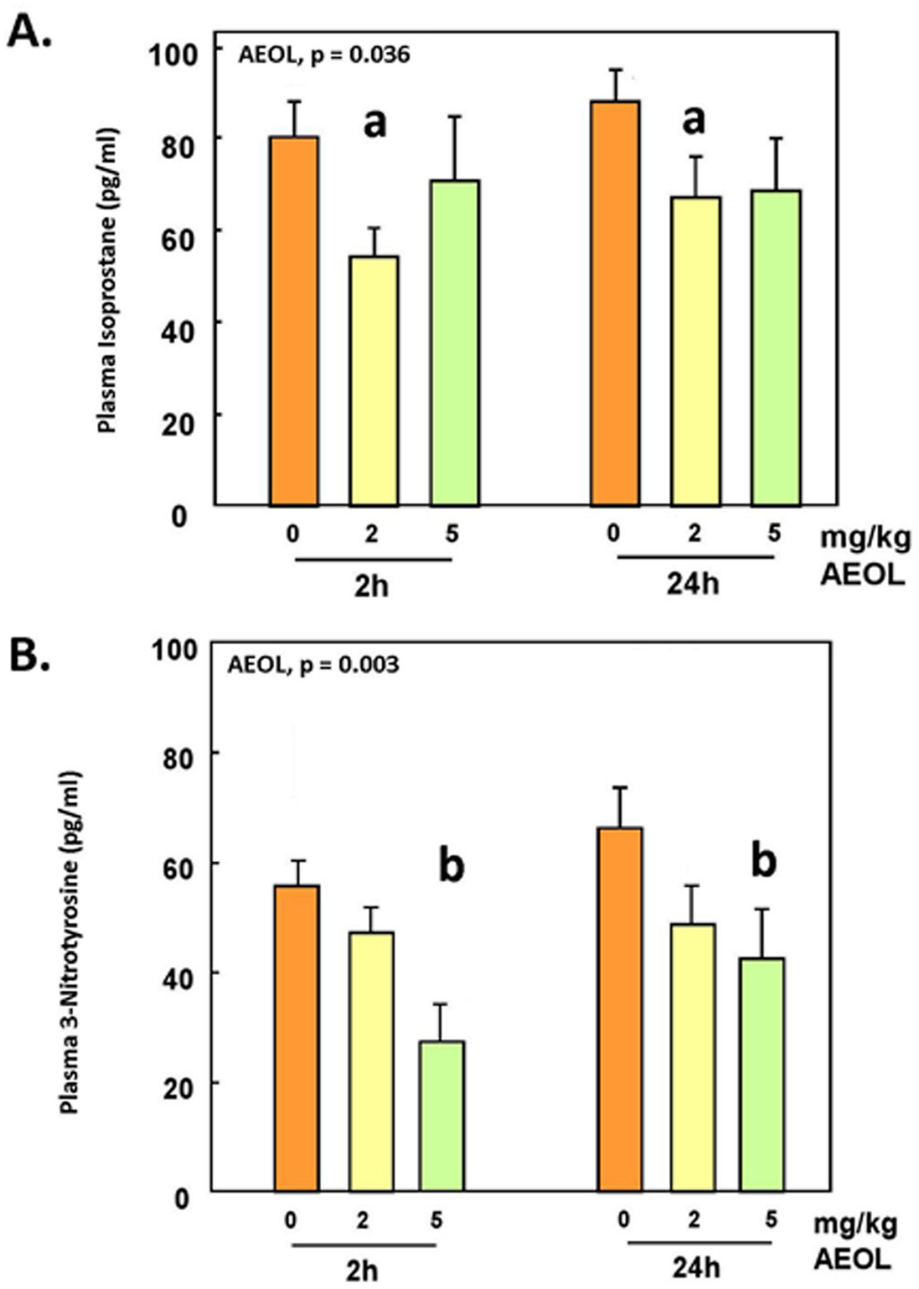
Plasma levels of isoprostane and 3-nitrotyrosine in animals treated with AEOL. Mean ± SE, n = 6 animals per group. **A**. Plasma isoprostane: Two-way ANOVA. AEOL main effect, p = 0.036. Holm-Sidak: **a**, 0 vs 2 mg/kg, p = 0.011. **B**. Plasma 3-nitrotyrosine: Two-way ANOVA. AEOL main effect, p = 0.003. Holm-Sidak: **b**, 0 vs 5 mg/kg, p < 0.001, and 2 vs 5 mg/kg, p < 0.040.

To confirm the antioxidant actions of AEOL 10150, we measured plasma levels of two markers of oxidative stress, 8-isoprostane and 3-nitrotyrosine. Levels of 8-isoprostane were significantly decreased (p<0.05) in animals injected with 2 mg/kg AEOL 10150 and tended to decrease at 5 mg/kg (not statistically significant) at both 2 and 24h post exposure (Fig 1A). 3-Nitrotyrosine exhibited a dose-dependent decrease (p<0.05) in rats injected with 2 and 5 mg/kg body weight AEOL 10150 (Fig 1B).

### Plasma Endothelin

**Fig 2:**
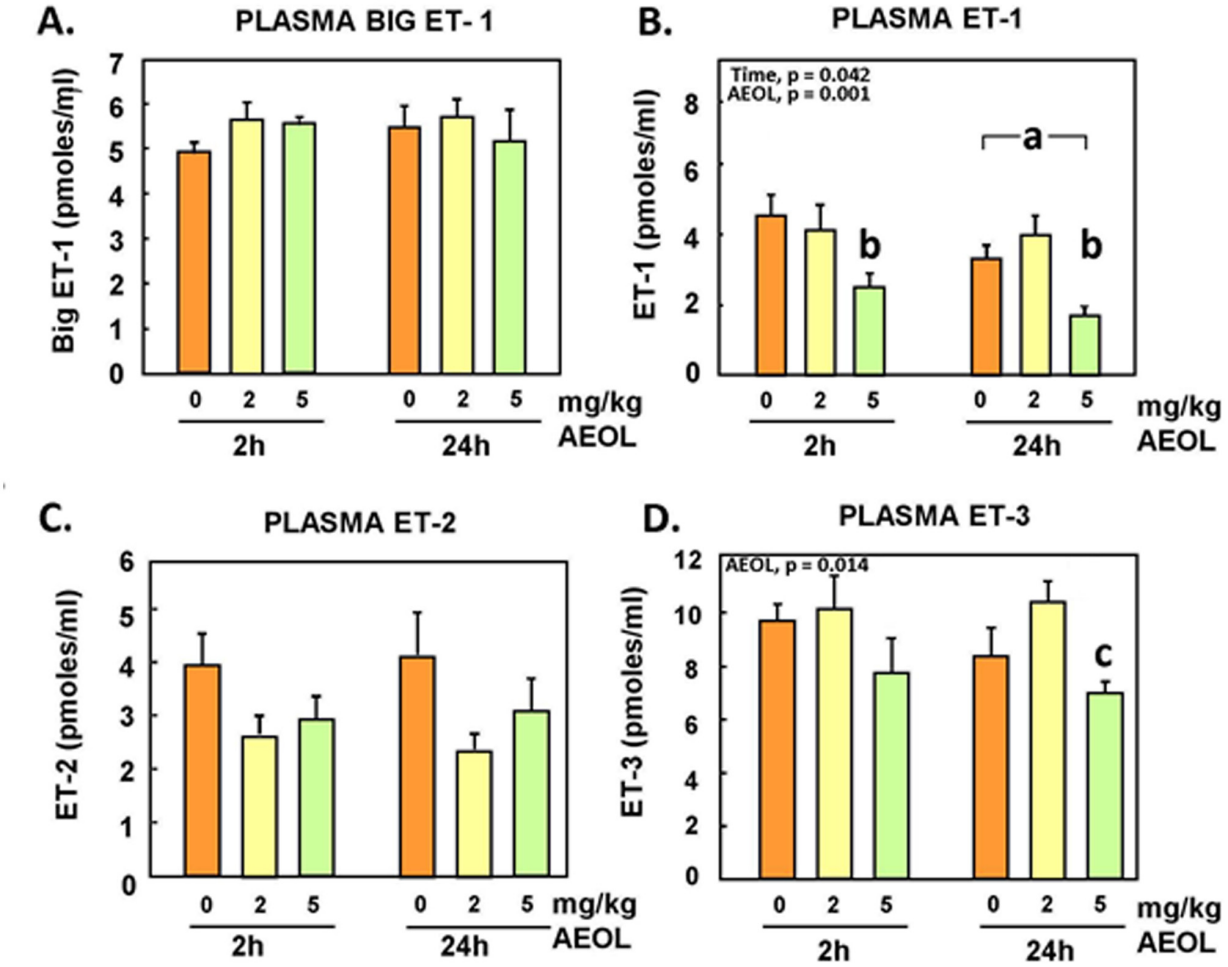
Plasma levels of bigET-1, ET-1, ET-2 and ET-3 peptides in animals treated with AEOL. Mean ± SE, n = 6 animals per group. **A.** BigET-1: NS. **B.** ET-1: Two-way ANOVA. TIME main effect, p = 0.042. Holm-Sidak: **a**, 2 h vs 24 h, p < 0.05. AEOL main effect, p = 0.001. Holm-Sidak: **b**, 0, 2 vs 5 mg/kg, p < 0.001. **C.** ET-2: NS. **D.** ET-3: Two-way ANOVA. AEOL main effect, p = 0.014. Holm-Sidak: **c**, 2 vs 5 mg/kg, p = 0.004.

Plasma endothelins (BigET-1, ET-1, ET-2, and ET-3) levels were analysed to investigate the impact of this antioxidant treatment on these vasoactive peptides. Plasma ET-1 and ET-3 levels were found to be decreased significantly (p<0.05) at the -5 mg/kg BW dose and at both 2h and 24h post-exposure (Fig 2). No statistically-significant changes in bigET-1 and ET-2 were detected, although treatment with AEOL 10150 tended to decrease plasma ET-2.

### Endothelin System Genes Expression in Lungs and Heart

**Fig 3:**
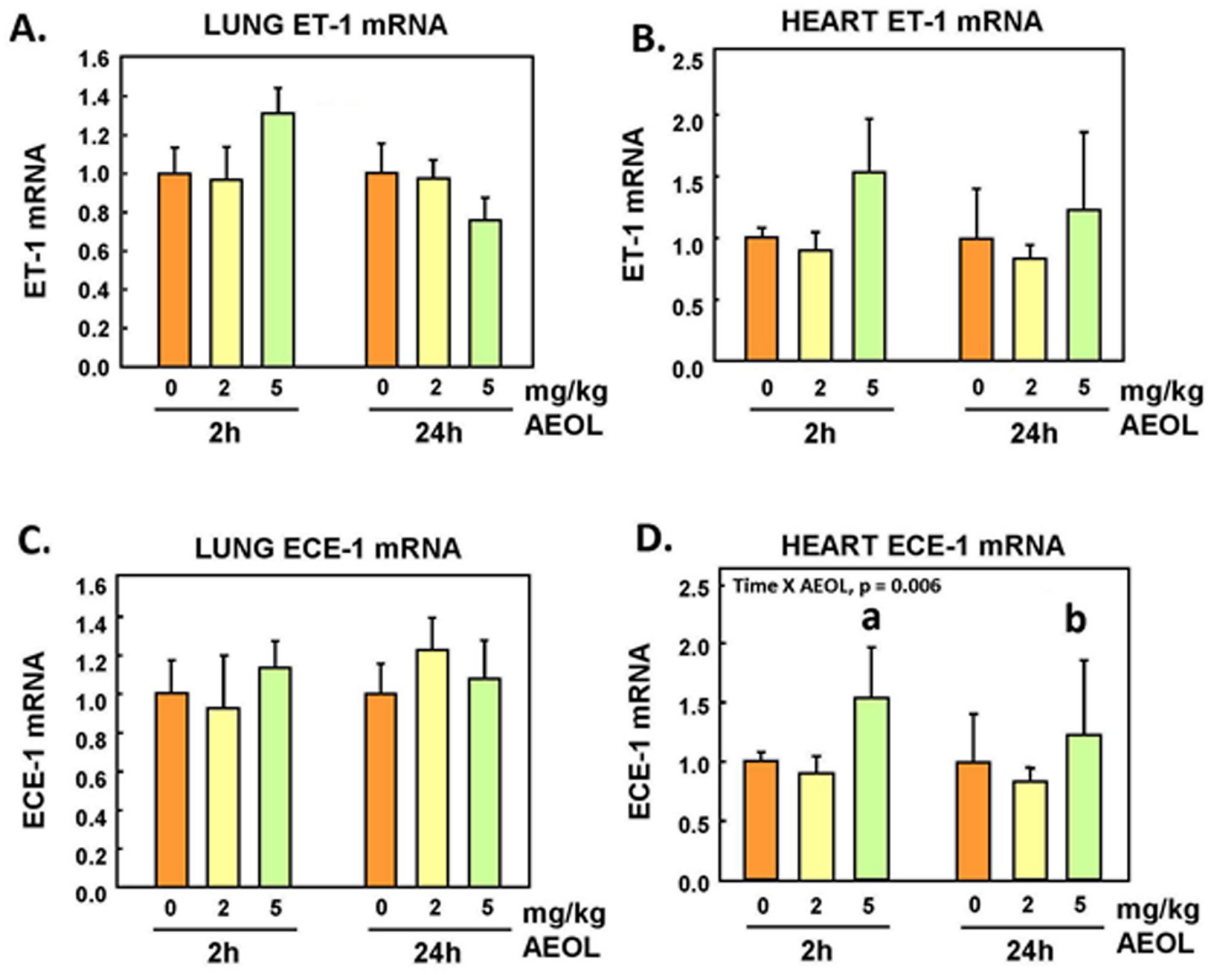
Lung and heart preproET-1 and ECE-1 mRNA. Mean ± SE, n = 6 animals per group. **A.** Lung preproET-1 mRNA: NS. **B.** Heart preproET-1 mRNA: NS. **C.** Lung ECE-1 mRNA: NS. **D.** Heart ECE-1 mRNA: Two-way ANOVA. TIME x AEOL interaction, p = 0.006. Holm-Sidak: **a**, AEOL within 2 h: 0, 2 vs 5 mg/kg, p<0.05. **b**, AEOL within 24 h: 2 vs 5 mg/kg, p = 0.029.

**Fig 4:**
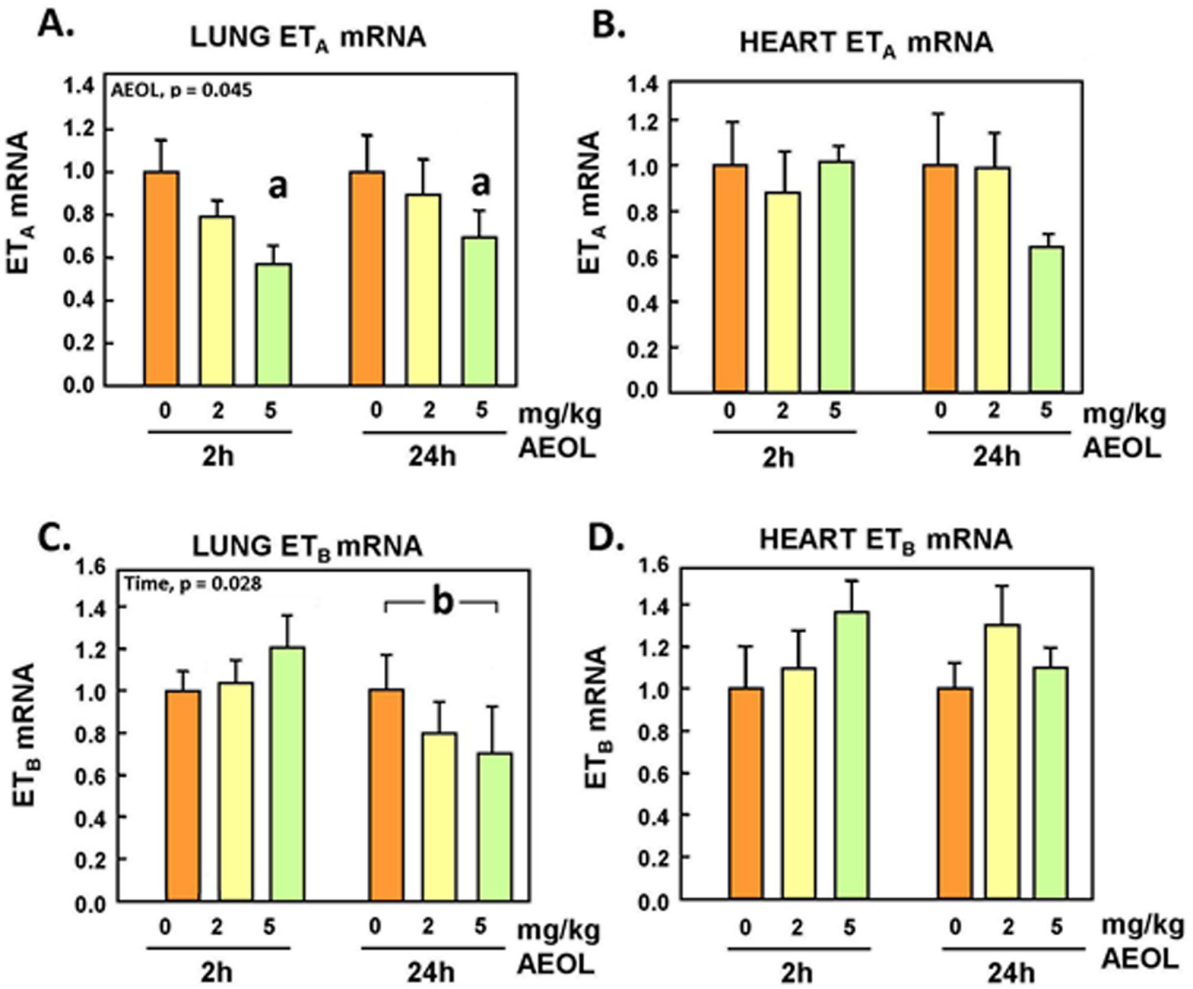
Lung and heart ET_A_ receptor and ET_B_ receptor mRNA. Mean ± SE, n = 6 animals per group. **A.** Lung ET_A_R. Two-way ANOVA. AEOL main effect, p = 0.045. Holm-Sidak: **a** 0 vs 5 mg/kg, p = 0.014. **B.** Heart ET_A_ mRNA: NS. **C.** Lung ET_B_R: Two-way ANOVA. TIME main effect, p = 0.028. Holm-Sidak: **b**, 2 vs 24 h, p < 0.05. **D.** Heart ET_B_ mRNA: NS.

Lung and heart preproET-1 mRNA levels exhibited an increasing trend 2h after exposure at the highest dose of AEOL 10150, but the changes did not reach statistical significance (Fig 3A and 3B). Endothelin converting enzyme (ECE)-1 mRNA expression was unchanged in the lung following AEOL 10150 injection (Fig 3C), but was increased (p<0.05) in the heart tissue 2h and 24h after treatment at 5 mg/kg (Fig 3D). Expression of the ET_A_ receptor mRNA was found to be significantly decreased (p<0.05) in the lungs of animals injected with 5 mg/kg dose of the drug at both 2h and 24h post exposure (Fig 4A), but not in the heart (Fig 4B). Meanwhile, ET_B_ receptor mRNA expression was significantly reduced (p<0.05) in the lungs of animals at 24h post-treatment (Fig 4A). There were no significant changes in heart ET_B_ receptor mRNA expression 2h and 24h after treatment with the drug (Fig 4B).

### eNOS and iNOS Gene Expressions in Lungs and Hearts

**Fig 5:**
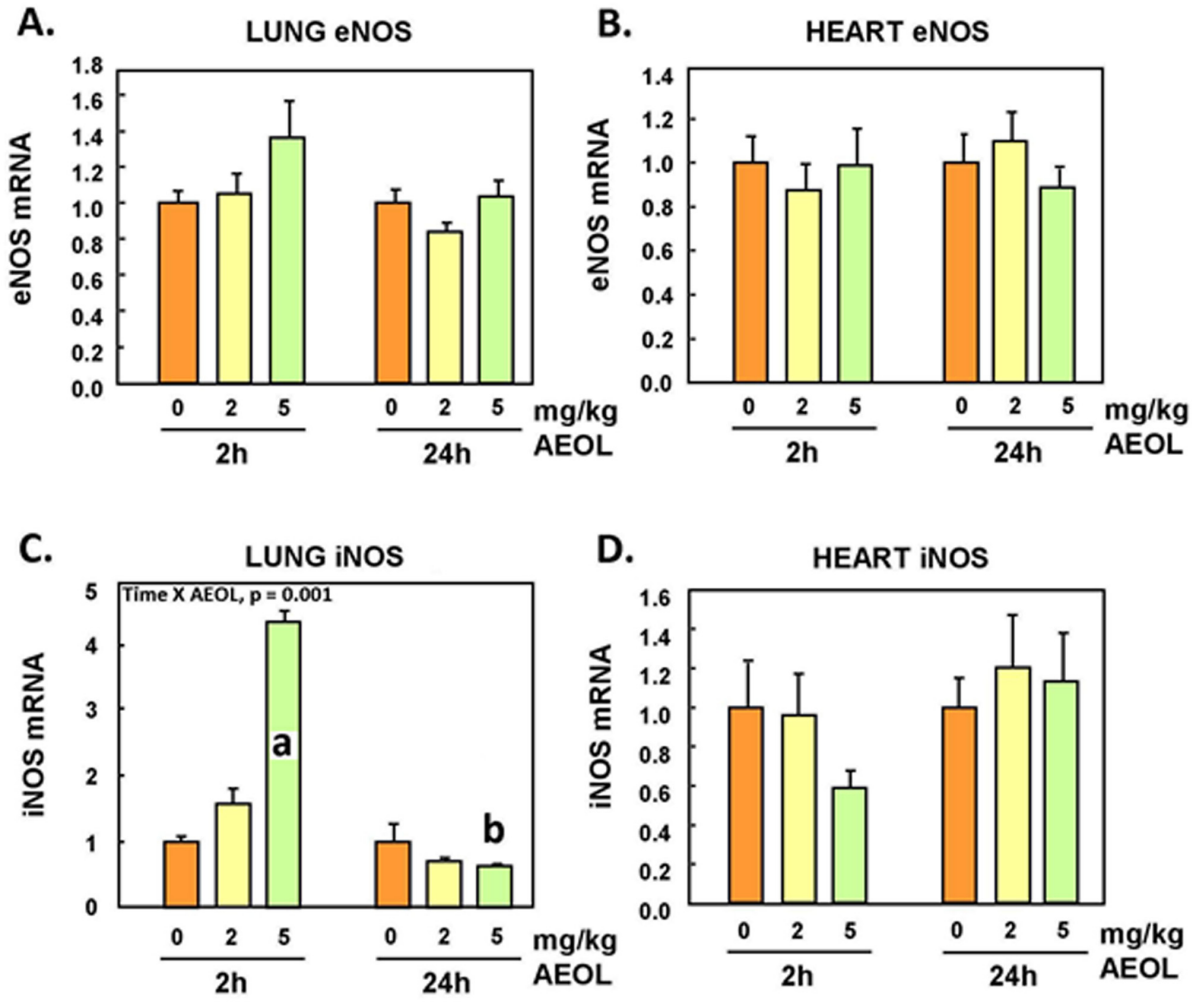
Lung and heart eNOS and iNOS mRNA. Mean ± SE, n = 6 animals per group. **A.** Lung eNOS: NS. **B.** Heart eNOS: NS. **C.** Lung iNOS: Two-way ANOVA. TIME x AEOL interaction, p < 0.001. Holm-Sidak: **a**, AEOL within 2 h: 0, 2 vs 5mg/kg, p < 0.001. **b**, AEOL within 24 h: 0 vs 5 mg/kg, p = 0.047. **D.** Heart iNOS: NS.

No statistically-significant changes in eNOS mRNA levels were observed in the lungs or heart (Fig 5A and 5B). However, lung iNOS mRNA expression significantly increased (p<0.05) at 5 mg/kg within 2h after injection of the drug but decreased afterward, down by 40% (p<0.05) 24 h post-treatment (Fig 5C). Changes of iNOS mRNA expression in the heart tissue were not statistically-significant (Fig 5D).

## Discussion

In the present study, the effect of the manganese superoxide dismutase mimetic AEOL 10150 on basal levels of oxidative stress in vivo and the endothelin system were examined in a healthy rat (Fischer 344) model. AEOL 10150 treatment led to decreased levels in plasma of 8-isoprostane levels, a product of lipid peroxidation, and 3-nitrotyrosine, a marker of oxido-nitrative stress. This confirms the role of this drug as an antioxidant and as a scavenger of superoxide anion that is known to participate in the formation of 8-isoprostane and 3-nitrotyrosine by generation of peroxynitrite species. Our observations are also consistent with the observation of a reduction of oxidative and nitrative stress by the superoxide dismutase mimetic tempol in models of diabetes [39, 40] or spinal cord injury [41].

The data revealed that treatment with AEOL 10150 decreased the circulating levels of the mature peptides ET-1 (Fig 3B) and ET-3 (Fig 3D) in a statistically-significant manner at the highest dose of 5mg/kg; ET-2 also displayed a trend to decrease but changes were not statistically-significant (Fig 3C). Plasma levels of the precursor peptide bigET-1 levels were not affected by treatment (Fig 2A), suggesting that decrease of systemic levels of circulating ET-1 was not due to decreased de novo synthesis. The lung vascular bed is a main source of circulating bigET-1 and ET-1, as well as the main site of receptor-mediated clearance of ET-1. Expressions of preproET-1 mRNA (Fig 3A) and ECE-1 mRNA (Fig 3C) in the lungs were not altered by the AEOL 10150 treatments, consistent with minimal impact of the drug on de novo synthesis of bigET-1 and processing of bigET-1 to ET-1 by ECE-1, at least in the lung parenchyma and vascular bed.

The ET_A_ receptor is known to enable vasoconstriction mechanisms in response to binding of mature endothelin peptides, with ET-1 being the most potent [42]. Binding to the ET_B_ receptor in the caveola of the lung capillary bed is the principal clearance mechanism of the mature ET-1 peptides; liganding of the ET_B_ receptor in most tissues also triggers NO synthesis and vasodilation responses [43]. Previous reports have shown that the loss of ET_B_ receptor led to increased circulating levels of ET-1 in vivo [44, 45]. Here, we show that expression of both ET_A_ and ET_B_ receptors mRNA expression were reduced 24 h after treatment of the animals with AEOL 10150 (Fig 4A and 4B). It may be that reduced oxidative stress is protective of the receptors during recycling, with decreased turnover and reduced requirement for de novo synthesis; however, the molecular and physiological significance of our observations remain to be established. Certainly, ET_B_/ET_A_ receptor antagonists are associated with a reduction in blood pressure [9, 46], illustrating the physiological relevance of reduced expression of endothelin receptors.

The lung, with its large vascular bed, is an important producer of vasoactive agents including vasoconstrictive endothelins and the vasodilatory molecule nitric oxide. In addition to the effects on endothelin receptor transcript levels, AEOL 10150 treatment resulted in an early increase in lung iNOS mRNA, within 2h after treatment, but followed by a 40% decrease over the following 24h. Interestingly, it has been reported that iNOS activity is essential for activation of antioxidant protection genes in rat aorta endothelial cells [47]. The human lung epithelial cells express iNOS and are responsible for most of the bronchodilating NO in exhalate [48]. Binding of ET-1 to ET_B_ receptors can lead to downstream signalling of vasodilation pathway through NO production [45], and it is possible that this is a favoured early pathway based on the observation of AEOL treatment-related increase of lung iNOS mRNA levels. A protective effect of iNOS on thrombosis in female mice has also been reported [49]. Nevertheless, despite early activation, iNOS expression was eventually decreased; it is also possible that AEOL 10150 treatment interfered with the corresponding downstream iNOS enzyme production and/or activity [50]. Both ET_B_ receptor and iNOS expression were observed to decline at 24h post AEOL exposure, which may be attributable to homeostatic gene expression mechanisms in response to higher levels of NO and reduced ET-1, as sustained NO production is metabolically toxic [51]. Overall, the 40% decrease of iNOS mRNA expression in the lungs, with the 25–30% decreases of isoprostane and 3-nitrotyrosine in plasma after AEOL treatment reported here are consistent with the observation of a reduction of oxidative and nitrative stress by the superoxide dismutase mimetic tempol in such models of diabetes [39, 40] or spinal cord injury [41].

Exposure to air pollutants that amplify oxidative stress increases transcript levels of ET genes in several organs and produces higher circulating ET levels [36, 52, 53]. Inhibition of the reactive oxygen species-generating enzyme xanthine oxidase with oxypurinol has been shown to reduce ET-1 expression and secretion in mammary arteries as well as in patients immediately following angioplasty [15]. Endothelin-1 plasma levels are also known to be elevated in hypertensive and diabetic patients, and correlate with lower antioxidant status and vitamin C concentrations [22]. In our present study, the decline in plasma markers of oxidative stress correlated with a marked decrease in plasma levels of ET-1 after treatment with AEOL 10150, consistent with the above stated association. As indicated earlier, there were no evidence here of changes in expression of preproET-1 or ECE-1 mRNA expression in the lungs, while the pulmonary expression the clearance receptor ET_B_ was actually reduced by 30% after treatment of the animals with AEOL 10150. Hence, it does not appear that reduction of circulating levels of ET-1 was due to downregulation of expression, decreased rate of maturation, or enhanced receptor-mediated clearance within the lungs. The decreases of circulating levels of ET-1 (p<0.05), ET-2 (NS) and ET-3 (p<0.05) could be due to reduced systemic gene expression of the prepropeptides or enhanced clearance by degradation. It is noteworthy that expressions of preproET-1 (NS) and ECE-1 (p<0.05) were increased in the heart after the higher dose of AEOL 10150. This could possibly represent a compensatory response of the heart to decreased circulating ET-1 that is aimed at maintaining the positive inotropic role of ET-1, which is in part regulated by superoxide flux [54, 55, 56].

Together, our data indicate that the catalytic antioxidant AEOL 10150 exerts effects on the endothelin/nitric oxide system in this healthy animal model. This is consistent with the reported hypotensive property of the drug [30] and a role for reactive oxygen species in the regulation of the endothelinergic system, in vasoconstriction and endothelial dysfunction.

## Conclusion

The present study demonstrates that the basal endogenous flux of reactive nitrogen species and reactive oxygen species regulate the homeostatic balance of the endothelin/nitric oxide system. Scavenging of free radicals by AEOL 10150 decreases baseline ET-1 levels likely through accelerated clearance, and may decrease sensitivity to ET-1 through down-regulation of ET_A_ receptor.

## Supporting Information

S1 ARRIVE Guidelines Checklist (PDF)

## References

[1] Khimji AK, Rockey DC (2010) Endothelin—biology and disease. Cell Signal 22: 1615–1625. 10.1016/j.cellsig.2010.05.002 20466059

[2] Hoffmann E, Assennato P, Donatelli M, Colletti I, Valenti TM (1998) Plasma endothelin-1 levels in patients with angina pectoris and normal coronary angiograms. Am Heart J 135: 684–688. 9539486 10.1016/s0002-8703(98)70286-8

[3] McMurray JJ, Ray SG, Abdullah I, Dargie HJ, Morton JJ (1992) Plasma endothelin in chronic heart failure. Circulation 85: 1374–1379. 1532540 10.1161/01.cir.85.4.1374

[4] Neuhold S, Huelsmann M, Strunk G, Struck J, Adlbrecht C, Gouya G et al (2010) Prognostic value of emerging neurohormones in chronic heart failure during optimization of heart failure-specific therapy. Clin Chem 56: 121–126. 10.1373/clinchem.2009.125856 19884490

[5] Nootens M, Kaufmann E, Rector T, Toher C, Judd D, Francis GS et al (1995) Neurohormonal activation in patients with right ventricular failure from pulmonary hypertension: relation to hemodynamic variables and endothelin levels. J Am Coll Cardiol 26: 1581–1585. 7594089 10.1016/0735-1097(95)00399-1

[6] Schumacher WA, Steinbacher TE, Allen GT, Ogletree ML (1990) Role of thromboxane receptor activation in the bronchospastic response to endothelin. Prostaglandins 40: 71–79. 2143841 10.1016/0090-6980(90)90057-3

[7] Stewart DJ, Levy RD, Cernacek P, Langleben D (1991) Increased plasma endothelin-1 in pulmonary hypertension: marker or mediator of disease? Ann Intern Med 114: 464–469. 1994793 10.7326/0003-4819-114-6-464

[8] Channick R, Badesch DB, Tapson VF, Simonneau G, Robbins I, Frost A et al (2001) Effects of the dual endothelin receptor antagonist bosentan in patients with pulmonary hypertension: a placebo-controlled study. J Heart Lung Transplant 20: 262–263. 10.1016/s1053-2498(00)00606-9 11250530

[9] Rubin LJ, Badesch DB, Barst RJ, Galie N, Black CM, Keogh A et al (2002) Bosentan therapy for pulmonary arterial hypertension. N Engl J Med 346: 896–903. 11907289 10.1056/NEJMoa012212

[10] Battistini B, Berthiaume N, Kelland NF, Webb DJ, Kohan DE (2006) Profile of past and current clinical trials involving endothelin receptor antagonists: the novel "-sentan" class of drug. Exp Biol Med (Maywood) 231: 653–695. 16740981

[11] Cowburn PJ, Cleland JG, McArthur JD, MacLean MR, McMurray JJ, Dargie HJ (1998) Short-term haemodynamic effects of BQ-123, a selective endothelin ET(A)-receptor antagonist, in chronic heart failure. Lancet 352: 201–202. 10.1016/S0140-6736(05)77807-7 9683214

[12] Kiowski W, Sutsch G, Hunziker P, Muller P, Kim J, Oechslin E et al (1995) Evidence for endothelin-1-mediated vasoconstriction in severe chronic heart failure. Lancet 346: 732–736. 7658874 10.1016/s0140-6736(95)91504-4

[13] Sakai S, Miyauchi T, Sakurai T, Yamaguchi I, Kobayashi M, Goto K et al (1996) Pulmonary hypertension caused by congestive heart failure is ameliorated by long-term application of an endothelin receptor antagonist. Increased expression of endothelin-1 messenger ribonucleic acid and endothelin-1-like immunoreactivity in the lung in congestive heart failure in rats. J Am Coll Cardiol 28: 1580–1588. 8917275 10.1016/s0735-1097(96)00336-1

[14] Kaehler J, Sill B, Koester R, Mittmann C, Orzechowski HD, Muenzel T et al (2002) Endothelin-1 mRNA and protein in vascular wall cells is increased by reactive oxygen species. Clin Sci (Lond) 103 Suppl 48: 176S–178S. 12193080 10.1042/CS103S176S

[15] Knappe D, Sill B, Tharun B, Koester R, Baldus S, Muenzel T et al (2007) Endothelin-1 in humans is increased by oxygen-derived radicals ex vivo and in vivo. J Investig Med 55: 306–314. 17963680 10.2310/6650.2007.00013

[16] Ruef J, Moser M, Kubler W, Bode C (2001) Induction of endothelin-1 expression by oxidative stress in vascular smooth muscle cells. Cardiovasc Pathol 10: 311–315. 11755377 10.1016/s1054-8807(01)00095-3

[17] Sen S, Chen S, Feng B, Wu Y, Lui E, Chakrabarti S (2011) American ginseng (Panax quinquefolius) prevents glucose-induced oxidative stress and associated endothelial abnormalities. Phytomedicine 18: 1110–1117. 10.1016/j.phymed.2011.06.013 21840692

[18] Pollock DM, Pollock JS (2005) Endothelin and oxidative stress in the vascular system. Curr Vasc Pharmacol 3: 365–367. 16248780 10.2174/157016105774329408

[19] Wedgwood S, Dettman RW, Black SM (2001) ET-1 stimulates pulmonary arterial smooth muscle cell proliferation via induction of reactive oxygen species. Am J Physiol Lung Cell Mol Physiol 281: L1058–L1067. 11597896 10.1152/ajplung.2001.281.5.L1058

[20] Dong F, Zhang X, Wold LE, Ren Q, Zhang Z, Ren J (2005) Endothelin-1 enhances oxidative stress, cell proliferation and reduces apoptosis in human umbilical vein endothelial cells: role of ETB receptor, NADPH oxidase and caveolin-1. Br J Pharmacol 145: 323–333. 15765100 10.1038/sj.bjp.0706193 PMC1576147

[21] Rodrigo R, Gonzalez J, Paoletto F (2011) The role of oxidative stress in the pathophysiology of hypertension. Hypertens Res 34: 431–440. 10.1038/hr.2010.264 21228777

[22] Skalska AB, Pietrzycka A, Stepniewski M (2009) Correlation of endothelin 1 plasma levels with plasma antioxidant capacity in elderly patients treated for hypertension. Clin Biochem 42: 358–364. 10.1016/j.clinbiochem.2008.11.002 19046960

[23] Day BJ, Crapo JD (1996) A metalloporphyrin superoxide dismutase mimetic protects against paraquat-induced lung injury in vivo. Toxicol Appl Pharmacol 140: 94–100. 8806874 10.1006/taap.1996.0201

[24] Kinnula VL, Crapo JD (2003) Superoxide dismutases in the lung and human lung diseases. Am J Respir Crit Care Med 167: 1600–1619. 12796054 10.1164/rccm.200212-1479SO

[25] Bowler RP, Arcaroli J, Abraham E, Patel M, Chang LY, Crapo JD (2003) Evidence for extracellular superoxide dismutase as a mediator of hemorrhage-induced lung injury. Am J Physiol Lung Cell Mol Physiol 284: L680–L687. 12618426 10.1152/ajplung.00191.2002

[26] Bowler RP, Sheng H, Enghild JJ, Pearlstein RD, Warner DS, Crapo JD (2002) A catalytic antioxidant (AEOL 10150) attenuates expression of inflammatory genes in stroke. Free Radic Biol Med 33: 1141–1152. 12374626 10.1016/s0891-5849(02)01008-0

[27] Smith KR, Uyeminami DL, Kodavanti UP, Crapo JD, Chang LY, Pinkerton KE (2002) Inhibition of tobacco smoke-induced lung inflammation by a catalytic antioxidant. Free Radic Biol Med 33: 1106–1114. 12374622 10.1016/s0891-5849(02)01003-1

[28] McGovern T, Day BJ, White CW, Powell WS, Martin JG (2011) AEOL10150: a novel therapeutic for rescue treatment after toxic gas lung injury. Free Radic Biol Med 50: 602–608. 10.1016/j.freeradbiomed.2010.12.001 21156205 PMC4026011

[29] O'Neill HC, Orlicky DJ, Hendry-Hofer TB, Loader JE, Day BJ, White CW (2011) Role of reactive oxygen and nitrogen species in olfactory epithelial injury by the sulfur mustard analogue 2-chloroethyl ethyl sulfide. Am J Respir Cell Mol Biol 45: 323–331. 10.1165/rcmb.2010-0214OC 21642592 PMC3175559

[30] Ross AD, Sheng H, Warner DS, Piantadosi CA, Batinic-Haberle I, Day BJ et al (2002) Hemodynamic effects of metalloporphyrin catalytic antioxidants: structure-activity relationships and species specificity. Free Radic Biol Med 33: 1657–1669. 12488134 10.1016/s0891-5849(02)01140-1

[31] Rabbani ZN, Salahuddin FK, Yarmolenko P, Batinic-Haberle I, Thrasher BA, Gauter-Fleckenstein B et al (2007) Low molecular weight catalytic metalloporphyrin antioxidant AEOL 10150 protects lungs from fractionated radiation. Free Radic Res 41: 1273–1282. 17957541 10.1080/10715760701689550

[32] Kumarathasan P, Goegan P, Vincent R (2001) An automated high-performance liquid chromatography fluorescence method for the analyses of endothelins in plasma samples. Anal Biochem 299: 37–44. 11726182 10.1006/abio.2001.5362

[33] Vincent R, Kumarathasan P, Goegan P, Bjarnason SG, Guenette J, Berube D et al (2001) Inhalation toxicology of urban ambient particulate matter: acute cardiovascular effects in rats. Res Rep Health Eff Inst 5–54. 11833973

[34] Bielecki A, Saravanabhavan G, Blais E, Vincent R, Kumarathasan P (2012) An efficient sample preparation method for high-throughput analysis of 15(S)-8-iso-PGF2alpha in plasma and urine by enzyme immunoassay. J Anal Toxicol 36: 595–600. 10.1093/jat/bks070 22989424 PMC3471526

[35] Kumarathasan P, Vincent R (2003) New approach to the simultaneous analysis of catecholamines and tyrosines in biological fluids. J Chromatogr A 987: 349–358. 12617061 10.1016/s0021-9673(02)01598-4

[36] Thomson E, Kumarathasan P, Goegan P, Aubin RA, Vincent R (2005) Differential regulation of the lung endothelin system by urban particulate matter and ozone. Toxicol Sci 88: 103–113. 16081523 10.1093/toxsci/kfi272

[37] Thomson E, Vincent R (2005) Reagent volume and plate bias in real-time polymerase chain reaction. Anal Biochem 337: 347–350. 15691517 10.1016/j.ab.2004.10.047

[38] Livak KJ, Schmittgen TD (2001) Analysis of relative gene expression data using real-time quantitative PCR and the 2(-Delta Delta C(T)) Method. Methods 25: 402–408. 11846609 10.1006/meth.2001.1262

[39] Rosales MA, Silva KC, Lopes de Faria JB, Lopes de Faria JM (2010) Exogenous SOD mimetic tempol ameliorates the early retinal changes reestablishing the redox status in diabetic hypertensive rats. Invest Ophthalmol Vis Sci 51: 4327–4336. 10.1167/iovs.09-4690 20335612

[40] Bourgoin F, Bachelard H, Badeau M, Larivière R, Nadeau A, Pitre M (2013) Effects of tempol on endothelial and vascular dysfunctions and insulin resistance induced by a high-fat and high-fructose diet in the rat. Can J Physiol 91: 547–561. 10.1139/cjpp-2012-0273 23826653

[41] Quan HH, Kang KS, Sohn YK, Li M (2013) Tempol reduces injury area in rat model of spinal cord contusion injury through suppression of iNOS and COX-2 expression. Neurol Sci 34: 1621–1628. 10.1007/s10072-013-1295-y 23354604

[42] Schneider MP, Boesen EI, Pollock DM (2007) Contrasting actions of endothelin ET(A) and ET(B) receptors in cardiovascular disease. Annu Rev Pharmacol Toxicol 47: 731–759. 17002597 10.1146/annurev.pharmtox.47.120505.105134 PMC2825895

[43] Sato K, Oka M, Hasunuma K, Ohnishi M, Sato K, Kira S (1995) Effects of separate and combined ETA and ETB blockade on ET-1-induced constriction in perfused rat lungs. Am J Physiol 269: L668–L672. 7491987 10.1152/ajplung.1995.269.5.L668

[44] Kelland NF, Bagnall AJ, Morecroft I, Gulliver-Sloan FH, Dempsie Y, Nilsen M et al (2010) Endothelial ET(B) limits vascular remodelling and development of pulmonary hypertension during hypoxia. J Vasc Res 47: 16–22. 10.1159/000231717 19672104

[45] Kelland NF, Kuc RE, McLean DL, Azfer A, Bagnall AJ, Gray GA et al (2010) Endothelial cell-specific ETB receptor knockout: autoradiographic and histological characterisation and crucial role in the clearance of endothelin-1. Can J Physiol Pharmacol 88: 644–651. 10.1139/Y10-041 20628430

[46] Galie N, Badesch D, Oudiz R, Simonneau G, McGoon MD, Keogh AM et al (2005) Ambrisentan therapy for pulmonary arterial hypertension. J Am Coll Cardiol 46: 529–535. 16053970 10.1016/j.jacc.2005.04.050

[47] Hemmrich K, Suschek CV, Lerzynski G, Kolb-Bachofen V (2003) iNOS activity is essential for endothelial stress gene expression protecting against oxidative damage. J Appl Physiol (1985) 95: 1937–1946. 12882997 10.1152/japplphysiol.00419.2003

[48] Mattila JT, Thomas AC (2014) Nitric oxide synthase: non-canonical expression patterns. Front Immunol 5: 478 10.3389/fimmu.2014.00478 25346730 PMC4191211

[49] Upmacis RK, Shen H, Benguigui LE, Lamon BD, Deeb RS, Hajjar KA et al (2011) Inducible nitric oxide synthase provides protection against injury-induced thrombosis in female mice. Am J Physiol Heart Circ Physiol 301: H617–H624. 10.1152/ajpheart.00667.2010 21602468 PMC3154673

[50] Pfeiffer S, Schrammel A, Koesling D, Schmidt K, Mayer B (1998) Molecular actions of a Mn(III)Porphyrin superoxide dismutase mimetic and peroxynitrite scavenger: reaction with nitric oxide and direct inhibition of NO synthase and soluble guanylyl cyclase. Mol Pharmacol 53: 795–800. 9547373 10.1124/mol.53.4.795

[51] Estevez AG, Sahawneh MA, Lange PS, Bae N, Egea M, Ratan RR (2006) Arginase 1 regulation of nitric oxide production is key to survival of trophic factor-deprived motor neurons. J Neurosci 26: 8512–8516. 16914676 10.1523/JNEUROSCI.0728-06.2006 PMC2570095

[52] Thomson E, Kumarathasan P, Vincent R (2006) Pulmonary expression of preproET-1 and preproET-3 mRNAs is altered reciprocally in rats after inhalation of air pollutants. Exp Biol Med (Maywood) 231: 979–984. 16741034

[53] Thomson EM, Kumarathasan P, Calderon-Garciduenas L, Vincent R (2007) Air pollution alters brain and pituitary endothelin-1 and inducible nitric oxide synthase gene expression. Environ Res 105: 224–233. 17662977 10.1016/j.envres.2007.06.005

[54] Kubin AM, Skoumal R, Tavi P, Konyi A, Perjes A, Leskinen H et al (2011). Role of reactive oxygen species in the regulation of cardiac contractility. J Mol Cell ardiol 50: 884–893. 10.1016/j.yjmcc.2011.02.005 21320508

[55] De Giusti VC, Correa MV, Villa-Abrille MC, Beltrano C, Yeves AM, de Cingolani GE et al (2008) The positive inotropic effect of endothelin-1 is mediated by mitochondrial reactive oxygen species. Life Sci. 83: 264–271. 10.1016/j.lfs.2008.06.008 18625248

[56] Cingolani HE, Villa-Abrille MC, Cornelli M, Nolly A, Ennis IL, Garciarena C et al (2006) The positive inotropic effect of angiotensin II: role of endothelin-1 and reactive oxygen species. Hypertension 47: 727–734. 16505203 10.1161/01.HYP.0000208302.62399.68

